# The Novel Property of Heptapeptide of Microcin C7 in Affecting the Cell Growth of *Escherichia coli*

**DOI:** 10.3390/molecules22030432

**Published:** 2017-03-08

**Authors:** Rensen Ran, Huan Zeng, Dong Zhao, Ruiyuan Liu, Xia Xu

**Affiliations:** 1 State Key Laboratory of Biochemical Engineering, Institute of Process Engineering, Chinese Academy of Sciences, Beijing 100190, China; sankeshumy@163.com (R.R.); liury928@163.com (R.L.); 2 School of Chemistry and Chemical Engineering, University of Chinese Academy of Sciences, Beijing 100049, China; 3 College of Chemical Engineering, Beijing University of Chemical Technology, Beijing 100029, China; zhxizai@163.com (H.Z.); zhaodong19920306@163.com (D.Z.)

**Keywords:** microcin C7, heptapeptide, cell growth, membrane integrity

## Abstract

Microcin C7 (McC), widely distributed in enterobacteria, is a promising antibiotic against antibiotic resistance. Previous studies have demonstrated that the heptapeptide of McC is only responsible for recognizing the inner membrane transporter *YejABEF* to deliver McC into microbial cells, but lacks the capacity for inhibiting microbial cell growth. In this study, the effect of the heptapeptide (MR) and two analogues, *N*-formylated heptapeptide (f-MR) and *N*-aceylated heptapeptide (a-MR), on microbial cell growth were examined. It is surprising to find that MR not only inhibits the activity of intracellular β-galactosidase, respiratory chain dehydrogenases, and 6-phosphogluconate dehydrogenases (6PGDH), but it is also able to inhibit *Escherichia coli* growth, and eventually leads to cell death at the lethal concentration of 5.34 mM within 10 min. The modification of MR results in a slight increase in the lethal concentration. Cell membrane integrity at the lethal concentration confirms that MR undergoes the inhibition effect, but not by destroying the cell membrane integrity. The novel property of MR provides a new insight into the Trojan horse strategy of McC and opens a new route for antibiotics design.

## 1. Introduction

Antibiotic resistance is a serious menace to global health in the 21st century [1], and may cause a death toll up to 10 million per year worldwide by 2050 [2]. Microcin C7 (McC), which is widely distributed in enterobacteria, is a small ribosomally-synthesized, potent antimicrobial peptide against a wide range of Gram-negative and some Gram-positive bacteria [3,4]. As a promising antibiotic against antibiotic resistance [5], numerous efforts have been made to understand the mechanism behind the McC antimicrobial activity [6,7].

**Figure 1:**
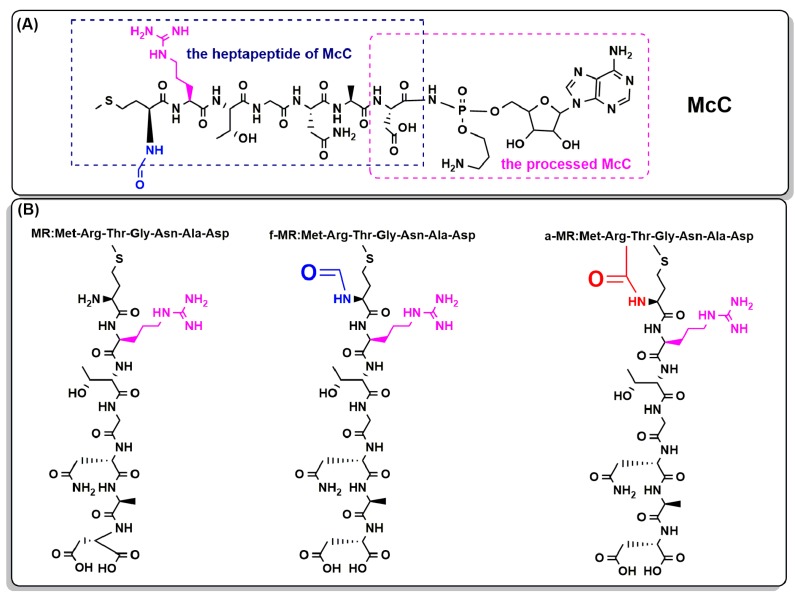
Chemical structures of McC, MR, and its analogues are shown. (**A**) The chemical structure of McC is represented; and (**B**) chemical structures of MR and f-MR, as well as a-MR, are shown.

McC, a Trojan horse antibacterial peptide [8,9], consists of a nonhydrolyzable aspartyl-adenylate conjugated to the heptapeptide of McC (MR) [3,9], as shown in Figure 1A. To kill bacteria, McC is actively transported into cells by MR through interacting with the ABC transporter *YejABEF* [10,11]. Once inside the cells, McC is metabolized by the peptide deformylase and aminopeptidases to liberate an analogue of nonhydrolyzable aspartyl adenylate with a propylamine [12,13,14]. Then, an adenosine triphosphate (ATP) and an aspartate (Asp) bind to the processed McC to inhibit aminoacylation of cognate tRNA^Asp^, leading to the blocking of protein synthesis and eventually resulting in cessation of translation and cell growth [15].

The previous studies on the antimicrodial mechanism of McC have demonstrated that the *N*-formylated heptapeptide of McC actually acts as a disguise to penetrate into target bacteria by recognizing the inner membrane transporter *YejABEF*, and the toxic entity is generated only when McC is inside target bacteria [14]. To distinguish the role of the heptapeptide in affecting *E. coli* growth, a few studies have been conducted in recent years. The peptide has been demonstrated to have no inhibition effects on the growth of *E. coli* at the concentration of 200 μM, although the peptide is able to inhibit (35*S*)-Met incorporation into newly-synthesized proteins in the coupled transcription-translation system at a 10-fold lower concentration [15,16]. Hence, MR, the hexapeptide carrier, is thoroughly regarded as a disguise only responsible for facilitating active import of McC into bacterial cells [9,12,13,14].

However, in this study, we accidentally found that MR is able to kill bacterial at a high concentration, implicating that MR is not only a signal peptide for transporting McC into cells but also has an ability to affect the growth of *E. coli* BL21, inconsistent with the previous study [15,16]. Hence, we tried to create a clear picture about how the presence of MR affects microbial cell growth. MR and its analogues, a-MR and f-MR, were constructed. The inhibition behavior of peptides acting on *E. coli* BL21 was evaluated using enzyme assays and scanning electron microscopy (SEM). The effect of permeabilizer compounds on cell growth was further detected. The novel property of MR may provide a new insight into the Trojan horse strategy of McC.

## 2. Results

### 2.1. Peptide Synthesis

**Figure 2:**
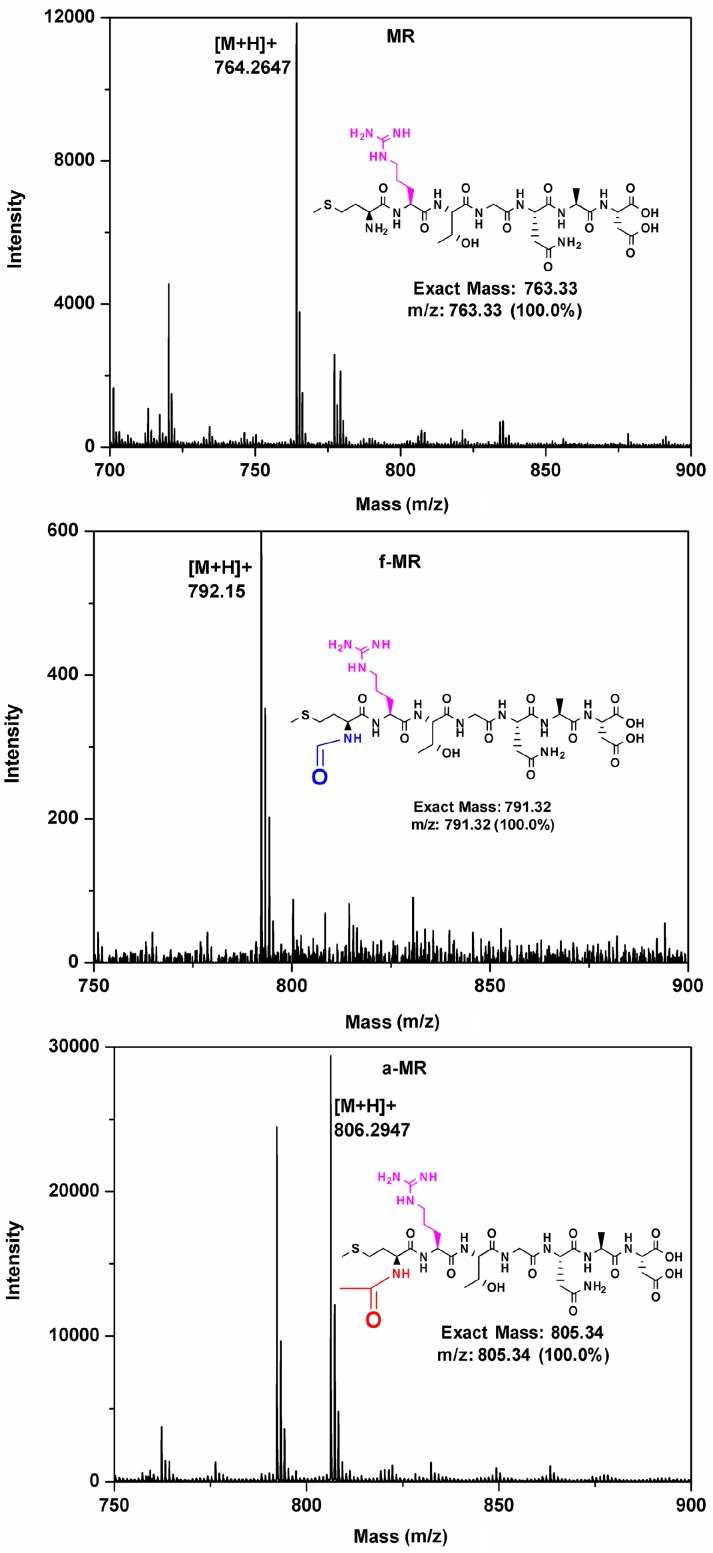
The MALDI-TOF MS analyses of peptides are shown.

To explore the effect of the *N*-modification on the property of hepapeptide, MR without the *N*-formyl group, f-MR with the *N*-formyl group, and a-MR with the *N*-aceyl group at the *N*-termini were synthesized and purified using reverse phase high-performance liquid chromatography (RP-HPLC). The products were analyzed by the matrix-assisted laser desorption/ionization time of flight mass spectrometry (MALDI-TOF-MS) to determine the molecular weight. As shown in Figure 2, the exact mass of MR, f-MR, and a-MR is 763.33, 791.32, and 805.34 Da, respectively. The hydrogenation peaks detected by mass spectrometry were consistent with their molecular weights individually.

### 2.2. Cell Growth

**Figure 3:**
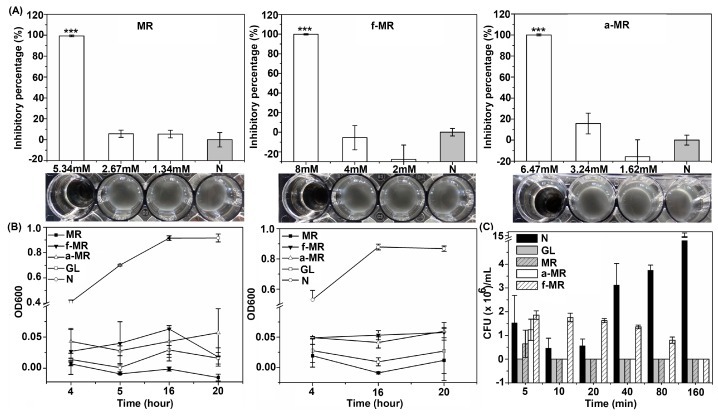
The cell growth of *E. coli* BL21 in the presence of peptides is shown. (**A**) The results of the broth macrodilution method used to determine the lethal concentration of peptides are shown. Results are mean values ± standard deviation (SD) of three independent experiments; (**B**) the OD600 of groups was changed along with time at their lethal concentration. Into each well of a 48-well plate was added 50 μL of each lethal concentration of peptides and 50 μL of *E. coli* BL21 (1 × 10^6^ CFU/mL) in LTM. The plate was incubated at 37 °C for 3 h. Then 0.9 mL MHB was added to incubate at 37 °C for 4, 5, 16, and 20 h. Ten microliters were then transferred from the lethal concentration wells to centrifuge tubes containing 2 mL MHB medium. The centrifuge tubes were incubated at 37 °C for 4, 16, and 20 h. GL is the positive control group at 2.1 mM. Results are mean values ± SD of three independent experiments; and (**C**) the result of killing kinetics assays is shown. MR and a-MR kill *E. coli* BL21 rapidly, while f-MR kills *E. coli* BL21 slowly. The bacteria were counted by CFU. Negative or positive controls were run without peptide or with GL. Results are mean values ± SD of three independent experiments. The symbol “N” represents the negative control group without peptides. Results are typical results of three separate experiments. *** α < 0.001 vs. control.

The cell growth of *E. coli* BL21 in the presence of MR, f-MR and a-MR was investigated using the macrodilution broth method. As shown in Figure 3A, the lethal concentration for MR, f-MR, and a-MR was 5.34, 8, and 6.47 mM, respectively. As indicated in Figure 3A, the modification of the MR peptide resulted in lower toxicity to *E. coli* BL21 than the MR peptide itself. To confirm that the bacteria were killed after being treated with peptides, 20 μL specimens from the treated groups were added into 2 mL fresh Muller-Hinton broth (MHB) medium and then cultured at 37 °C for 20 h. As shown in Figure 3B, there were no viable bacteria which existed in the medium with the similar pattern as the positive anoplin (GL) peptide control group, which holds a bactericidal activity [17,18,19]. From the killing kinetic experiments, it was further found that MR at 5.34 mM and a-MR at 6.47 mM were able to kill the bacteria (3log_10_ killed) within 10 min while the f-MR at 8 mM killed the bacteria within 160 min (Figure 3C).

### 2.3. Media Effect

**Figure 4:**
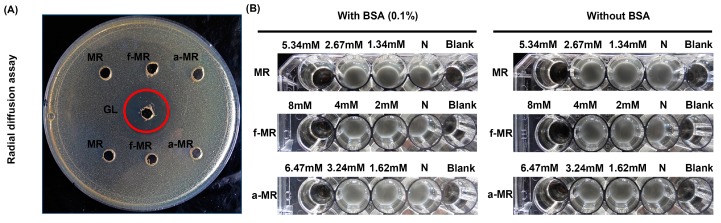
The media effect on the activity of peptides is shown. (**A**) The result of radial diffusion assay is shown. All of MR, f-MR, and a-MR was at 10 mM, while GL was at 2.1 mM; and (**B**) the antibacterial activity of peptides with 0.1% BSA in the LTM is shown. The symbol “N” represents the negative control group without peptides. Results are typical results of three separate experiments.

As demonstrated in Figure 3, the cell growth of bacteria was inhibited by the presence of the peptides, and completely killed at the lethal concentration when cultured in the MHB culture. However, when using the radial diffusion assay, there was no inhibition zone observed for all peptides, even at the high concentration of up to 10 mM (Figure 4A). Additionally, we examined the effect of bovine serum albumin (BSA) in the culture medium on the cell growth in the presence of the peptides. As seen in Figure 4B, the lethal concentration remained the same as that in the absence of BSA.

### 2.4. Enzyme Activity

**Figure 5:**
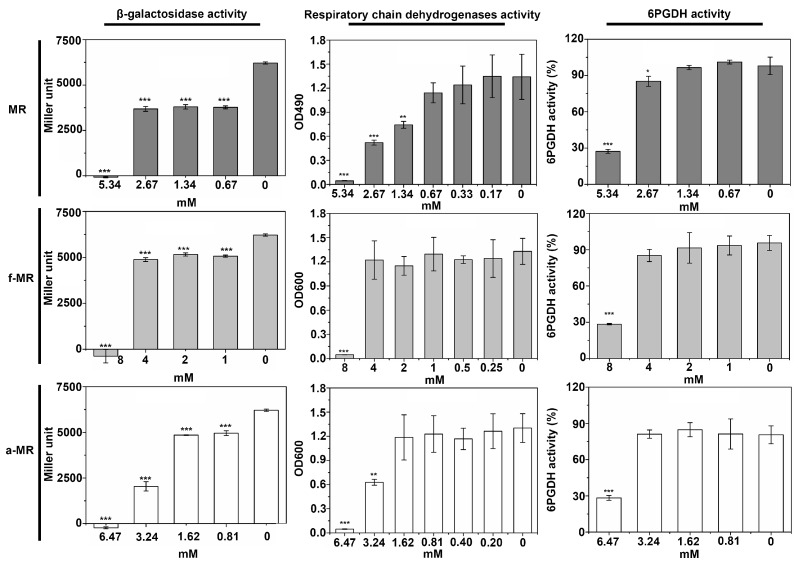
The results of enzyme activity assays are shown. The same amount of bacteria without peptide was used as a negative control, dubbed “0”. The symbol * and **, as well as *** represent that there is a significant difference between the testing groups and the negative group at α < 0.05 and α < 0.01, as well as α < 0.001, respectively. Results are mean values ± SD of three independent experiments. * α < 0.05, ** α < 0.01, *** α < 0.001 vs. control.

To determine how the peptides affect the cell growth, the effects of the peptides on the activity of β-galactosidase, respiration chain dehydrogenases, and 6PGDH were evaluated. β-galactosidase, the enzyme responsible for the first step in the breakdown of lactose to galactose and glucose, is a key provider in the production of energy and a source of carbons. The activity of respiratory chain dehydrogenase, the constitutive enzymes of *E. coli*, is an indicator of its expression [20]. 6PGDH, which can convert the 6-phophogluconate and oxidized nicotinamide adenine dinucleotide (NAD^+^) to the reduced nicotinamide adenine dinucleotide (NADH), belongs to the constitutive enzyme in the respiratory chain dehydrogenase [21]. As shown in Figure 5, the trend in the activity of all tested enzymes was quite similar. A gradual decrease in the enzyme activity was detected when the peptide concentration of MR, f-MR, and a-MR increased, falling nearly to zero when the concentration of the peptides was at the lethal concentration.

### 2.5. Cell Morphology

**Figure 6:**
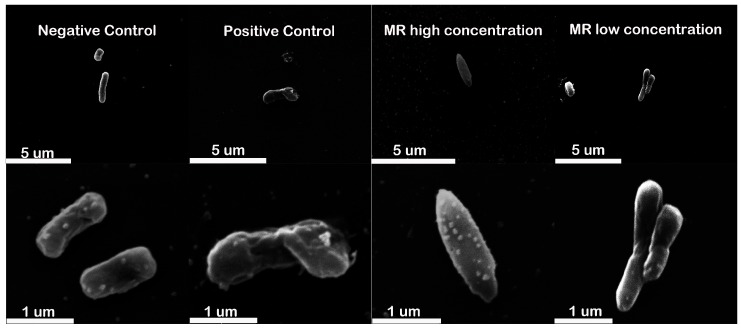
The cell morphology of the peptide-treated *E. coli* BL21 is shown. Negative control group: *E. coli* BL21 was treated without peptides. Positive control groups: *E. coli* BL21 was treated with GL at twice of the lethal concentration for up to 60 min. *E. coli* BL21 was treated with MR at twice of the 5.34 mM for up to 60 min, dubbed “MR high concentration”. *E. coli* BL21 was treated with MR at 0.05 mM for up to 60 min, dubbed “MR low concentration”. Results are typical results of three separate experiments. Approximately 2 × 10^7^ cells (in 50 μL) were transferred into a 1.5-mL Eppendorf tube and the peptide was added at the desired concentration.

Cell morphology of *E. coli* BL21 treated with the sublethal and lethal concentrations of peptides were detected using the SEM. The cells treated with GL were used as the positive control. Untreated *E. coli* cells displayed a smooth, bright surface with a plump rod shape, while the GL-exposed cells exhibited deep roughening of cell surface and a collapsed cell structure (Figure 6). Compared with the negative control, there was a dramatic change in the cell morphology from the initial rod shape to the “fusiform” shape when the MR concentration increasing to the lethal concentration (Figure 6). However, no deep roughness was observed.

### 2.6. Membrane Integrity

**Figure 7:**
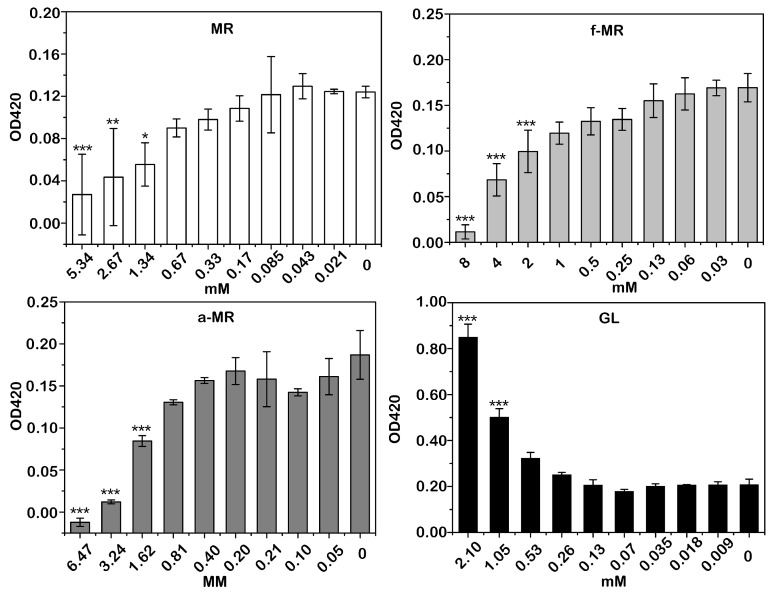
The membrane integrity of peptide-treated *E. coli* BL21 is shown. The same amount of bacteria without peptide was used as a negative control, dubbed “0”, and the positive control was run with different concentrations of GL. Results are the mean values ± SD of three independent experiments. * α < 0.05, ** α < 0.01, *** α < 0.001 vs. control.

β-galactosidase, an intracellular enzyme, is often used to monitor the integrity of the inner cell membrane [22]. As shown in Figure 7, a totally different trend in the changes of the β-galactosidase activity was observed. The β-galactosidase activity was increased with the GL concentration, while the β-galactosidase activity was decreased along with the increasing concentration of the other peptides tested here. 

### 2.7. Effects of Outer Membrane Permeabilization Agents

**Figure 8:**
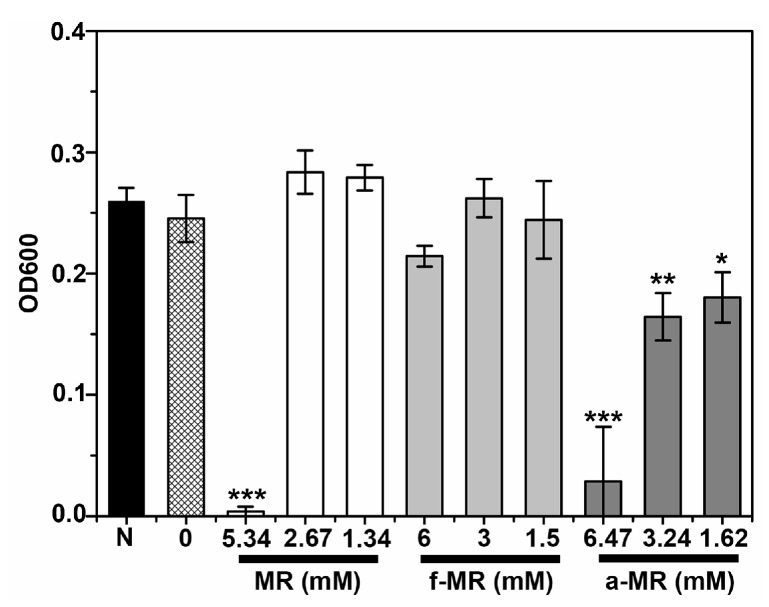
Effects of outer membrane permeabilization agents on the antibacterial activity of peptides are shown. The “0” mark represents the group with 0.05 mM EDTA and 0.35 mM Tris, but without peptides, while the “N” mark represents the group without the agent and peptides as the negative group. The rest of the group was mixed with 0.05 mM EDTA and 0.35 mM Tris, as well as peptides at the different concentrations. Results are the mean values ± SD of three independent experiments. * α < 0.05, ** α < 0.01, *** α < 0.001 vs. control.

A number of outer membrane permeabilization agents interacting with lipopolysaccharide (LPS) have been proved to enhance antibiotic drug uptake and further improve the antibacterial activity of the antimicrobial peptides [23,24,25]. Hence, to investigate the effects of outer membrane permeability changes on the effect of MR, f-MR, and a-MR on the cell growth, the outer membrane permeabilizing agents (EDTA and Tris) were used in this study. As shown in Figure 8, the lethal concentration for all three peptides was at the same level as that without using EDTA and Tris.

## 3. Discussion

McC is a promising antibiotic against antibiotic resistance. Apart from the delivery of McC into microbial cells through recognizing the inner membrane transporter *YejABEF*, a novel property of MR for killing bacteria was found in this study. To elucidate the detailed information about this novel property, three peptides were constructed, including MR, the *N*-formyl group modified peptide of f-MR and the *N*-acetyl group modified peptide of a-MR. The effects of the peptides on the cell growth, enzyme activity, cell morphology, and cell membrane integrity were detected. 

McC is delivered into bacterial cells with the help of MR through the ABC transporter *YejABEF* on the cell membrane [10,11]. As previously reported, the f-MR and MR peptides can inhibit the protein synthesis in vitro but not affect the cellular growth [15,16]. However, in this study, MR, and its analogues, have been demonstrated to have the capacity of leading to cell death, inconsistent with the previous studies [15,16]. One reason for this could be the high concentration used here, compared to 200 μM in the previous study [15]. Additionally, the difference in the lethal concentration between MR and f-MR indicates that the N terminal modification has effects on the antibacterial activity, as reported by Olga et al. [26,27].

In order to prove that MR has the capacity of killing *E. coli*, it is critical to minimize interference from the component in the medium [28,29]. Hence, in the macrodilution broth method, the key step is to mix microorganisms with the peptide in minimal medium [29,30]. In this study, the peptides of MR, f-MR, and a-MR were found to kill *E. coli* at the high concentration when the liquid testing medium (LTM) containing 1% MHB was used. However, there was no inhibition effect on *E. coli* growth when a rich nutrient system containing some molecules, such as mucins or nucleic acids, was employed (Figure 4A). These molecules may bind peptides and suppress their antimicrobial activity [28]. In addition, the difference in the culture medium used in assessing the effect of MR on cell growth could be another reason for the inconsistency between our results and the previous studies. MR, a recognition sequence for maturation machineries [4], is able to inhibit the protein synthesis, but not able to kill bacterial cells [15,16]. However, the experimental results show that the reduction in the enzyme activity with the increasing peptide concentration eventually leads to the cell death at the lethal concentration. The inhibition effect of the modified MR also implicates that the modification does not affect the interaction with the ABC transporter *YejABEF* and can be translocated into cells.

To determine the integrity of the inner membrane in the presence of peptides, the β-galactosidase activity, an indicator for the integrity of the membrane [22,31], was detected when using the OPNG method in the absence of permeabilization agents. As shown in Supporting Information Figure S1, there was a basal value of β-galactosidase activity for the cells cultured in the normal culture medium. The increase in the β-galactosidase activity from the basal value at the high GL concentration indicates the integrity loss of the inner membrane, consistent with the previous report [32]. However, when the cells were exposed to the high MR concentration, the β-galactosidase activity decreased from the basal value (Figure 7), illustrating that the cell membrane remains integral, which was also confirmed by the SEM results (Figure 6), indicating that the cell death is not caused by the cell membrane damage. Taking account of the number of colony formation units at the different concentrations of peptides in Figure S2, the presence of MR led to the reduction of enzyme activity, and eventually resulted in the death of *E.coli*.

The outer cell membrane can significantly slow the influx and uptake of antibiotics [23,24,25,33,34]. A number of outer membrane permeabilizing agents, such as EDTA and Tris, are known to interact with lipopolysaccharides (LPS) causing permeabilization of the outer membrane to enhance antibiotic drug uptakes leading to an improvement of antimicrobial activity [24,35]. Actually, we evaluated the effect of changes caused by the presence of permeabilizing agents (EDTA and Tris) in the outer membrane permeability on the lethal concentration of MR and its derivatives. According to the references, we decided to use 0.05 mM EDTA and 0.35 mM Tris, which are sufficient to cause membrane permeability changes [25,36,37]. The experimental results reveal that the changes in the outer membrane permeability does not lead to the changes in the lethal concentration of the peptides (Figure 8), indicating that the uptake of peptides is dependent on the interaction between the peptide and the ABC transporter *YejABEF*, not controlled by the outer membrane permeability.

## 4. Materials and Methods

### 4.1. Materials

Fluoren-9-ylmethoxycarbonyl (Fmoc) amino acid derivatives with side-chain protections were purchased from the GL Biochem Co., Ltd. (Shanghai, China). 2-Nitrophenyl β-d-galactopyranoside (ONPG), oxidized nicotinamide adenine dinucleotide (NAD^+^), 6-phosphogluconate, iodonitrotetrazolium chloride (INT) and reagents for peptide synthesis were purchased from the Aladdin Industrial Corporation (Shanghai, China). Regents about ethylenediaminetetraacetic acid (EDTA), sodium deoxycholate, hexadecyltrimethylammonium bromide (CTAB), beta-mercaptoethanol, sodium salts, and dimethylformamide (DMF) were obtained from the Sinopharm Chemical Reagent Co., Ltd. (Beijing, China). Muller-Hinton Broth (MHB) was purchased from the Beijing Ao Bo Xing Bio-Tech (Beijing, China). Agar powder and Tris were purchased from Gene Company Co., Ltd. (Hong Kong, China). ZoRBAX SB-C18 PreHT Column was purchased from the Agilent Technologies Inc. (Santa Clara, CA, USA). Acetonitrile with HPLC grade was purchased from the Thermo Fisher Scientific Inc. (Shanghai, China). Bovine Serum Albumin (BSA) was purchased from Beijing Solarbio Science and Technology Co., Ltd. (Beijing, China).

### 4.2. Peptide Synthesis and Purification

Three peptides of f-MR, MR, and a-MR, as shown in Figure 1B, were synthesized using the solid-phase synthesis strategy [38]. Briefly, Fmoc-6-aminocaproic acid (0.3 mmol/g resin) was firstly coupled to the Wang resins in the reaction mixture of *N*,*N*,*N'*,*N'*-tetramethyl-*O*-(benzotriazol-1-yl)uronium tetrafluoroborate (0.91 g), 0.52 mL ethyldiisopropylamine and 1-hydroxybenzotriazole (0.45 g) in 10 mL DMF at room temperature for 2 h. To deprotect the *N*-terminal Fmoc group, 5 mL of 20% piperidine in DMF was added to the resins for 30 min. Then other Fmoc amino acids were introduced in sequence. After synthesis, the product was cleaved using a cleavage cocktail of 10 mL trifluoroacetic acid/triisopropylsilane/1,2-ethanedithiol/water with the volume ratio of 94:1:2.5:2.5 (*v/v/v/v*) for 120 min at room temperature with gentle stirring. The cleavage solution was filtered and then evaporated under vacuum at room temperature. Compounds were purified using a RP-HPLC (LC-6AD) (Shimadzu Corporation, Kyoto, Japan) on a C18 column with a linear acetonitrile/water (0.1% TFA) gradient. Mass spectrometry (MALDI-TOF-TOF 4800 Plus) (ABI, Carlsbad, CA, USA) was used to determine the molecular weight of the purified products. The peptides with purity greater than 97% were used for further experiments.

### 4.3. Cell Growth

To determine the lethal concentration of peptides that prevent visible growth of bacteria, the macrodilution broth method was used [39,40,41]. Briefly, *E. coli* BL21 was cultured in MHB medium at 37 °C. Peptides were dissolved in the liquid testing medium (LTM) containing 10 mL of 100 mM phosphate buffer, 1 mL MHB, 2 mL 5 M NaCl in 87 mL deionized water at pH 6.5 as a stock solution. 50 μL of *E. coli* BL21 (1 × 10^6^ CFU/mL, colony forming units) and 50 μL of desired concentration of peptides in the LTM were transferred to a 48-well plate, then incubated at 37 °C with a shaking speed at 80 rpm for 3 h. Then 0.9 mL of MHB was added to the well and incubated without aeration at 150 rpm for 16–18 h at 37 °C. To determine the activity of the peptides, 10 μL of *E. coli* BL21 from the lethal concentration wells was transferred to a centrifuge tube containing 2 mL MHB medium and incubated at 37 °C for 16–18 h. The OD600 of *E. coli* BL21 solutions was measured at 600 nm using a spectrophotometer (Shanghai Spectrum Instruments Co., Ltd., Shanghai, China). The effect of bovine serum albumin (BSA) on the antibacterial activity was detected when 0.1% BAS (*w/w*) was present in the LTM. *E. coli* cultured without peptides was used as the negative control, and *E. coli* cultured with the anoplin peptide (GL) was used as the positive control. The calculation of the inhibitory percentage is as follows:

Inhibitory percentage = 100% × [1 − (OD600 of sample/OD600 of negative control)]
(1)

To evaluate the effects of permeabilizer compounds on the antimicrobial activity of peptides (MR, f-MR, a-MR), instead of LTM, the LTM containing 0.05 mM EDTA and 0.35 mM Tris was used.

### 4.4. Kill Time Assay

The killing kinetics of the peptides against *E. coli* BL21 was determined by counting the colony forming units (CFU) of live bacteria with agar plating [42]. The *E. coli* cells were incubated at 37 °C in the presence of peptides at the lethal concentration. The serial dilution strategy was carried out before the cells were plated out in Muller-Hinton agar (MHA) plates at predetermined time intervals (0, 5, 10, 20, 40, 80, and 160 min). The number of CFU was counted after overnight incubation at 37 °C.

### 4.5. Enzyme Activity Assays

β-galactosidase activity assay [22,43]: To determine the presence of peptides on the effect of β-galactosidase activity, permeabilization agents (100 mM dibasic sodium phosphate, 20 mM KCl, 2 mM MgSO_4_, 0.8 mg/mL CTAB, 0.4 mg/mL sodium deoxycholate, 5.4 μL/mL β-mercaptoethanol) was introduced to the cell solution before the addition of ONPG. As described previously [24], *E. coli* BL21 was cultured in the MHB medium supplemented with 1% lactose at 37 °C until OD600 reached ~0.5. Then the bacterial solution was diluted to 1–2 × 10^8^ CFU/mL using the LTM containing 0.01% lactose, as required by the McFarland 0.5 standard. Five hundred microliters of the diluted bacterial solution was incubated with 200 μL of peptides at different concentrations for 60 min at 30 °C and then filtered through a 0.22 μm filter. ONPG (60 mM Na_2_HPO_4_, 40 mM NaH_2_PO_4_, 1 mg/mL ONPG, 2.7 μL/mL β-mercaptoethanol) was introduced to the filtered solution [24]. To examine the integrity of the cell membrane, the β-galactosidase activity was measured using ONPG, described above, in the absence of permeabilization agents. The hydrolyzate of ONPG was recorded at 420 nm using a spectrophotometer. The calculation of the Miller unit is as follows:

Miller unit = 1000 × [OD420 − (1.75 × OD550)]/(T × V × OD600)
(2)

where T = reaction time in minutes; V = volume of culture assayed in milliliters; OD550 is the scatter from cell debris, which, when multiplied by 1.75 approximates the scatter observed at 420 nm.

Respiratory chain dehydrogenases activity assay: After 1 h of incubation with the desired concentration of peptides at 37 °C, the INT solution (0.1 mL 0.5%) was added to 100 µL bacterial solution and incubated at 37 °C in the dark for 2 h before adding 50 μL formaldehyde to terminate the reaction. To collect the production, the solution was centrifuged at 13,680× *g* for 10 min and then distilled twice using 250 μL of 50% (*v/v*) acetone in ethanol. The absorbance of supernatants was measured at 490 nm using a spectrophotometer (Shanghai Spectrum Instruments Co., Ltd., Shanghai, China) [44]. 

6PGDH activity assay: Cell extracts obtained from exponentially-growing cultures were added to 100 mM HEPES buffer (pH 7.5) containing 2 mM 6-phosphogluconate, 2 mM NAD^+^, 5 mM MgCl_2_ and 0.5 mM MnCl_2_ at 37 °C for 30 min. The conversion of NAD^+^ to NADH was monitored at 340 nm using a spectrophotometer (Shanghai Spectrum Instruments Co., Ltd., Shanghai, China) [45,46]. The calculation of the 6PGDH activity is as follows:

6PGDH activity = 100% × (OD340 of sample/OD340 of negative control)
(3)

### 4.6. Scanning Electron Microscopy 

As described previously [47], *E. coli* BL21 cells were harvested during exponential phase using centrifugation at 3000× *g* for 20 min, washed twice with 10 mM sodium phosphate buffer, and then resuspended in the same buffer. To immobilize bacterial cells, the cells were placed on a polylysine-coated glass slide, incubated at 30 °C for 90 min. The cells were then fixed with 2.5% (*w/v*) glutaraldehyde in 0.1 M sodium phosphate buffer, followed by washing with the same buffer and, finally, dehydrated with a series of graded ethanol treatments. After lyophilization with *tert*-butanol and gold coating, the samples were observed with scanning electron microscopy (FEI Quanta 200) (FEI, Eindhoven, The Netherlands).

### 4.7. Statistical Analysis 

Unless indicated otherwise, each experiment was performed in triplicate. The *t-*test (* α < 0.05, ** α < 0.01, *** α < 0.001) was applied to determine the statistical significance using Statistix 9 software. 

## 5. Conclusions

In this paper, we investigated the effects of MR and its derivatives, f-MR and a-MR, on cell growth. It was found that MR can inhibit the activity of β-galactosidase, respiration chain dehydrogenases, and 6PGDH and further leads to the cell death without damaging the inner cell membrane at the lethal concentration. The modification at the *N*-terminal has effects on the activity of the peptides. This novel property of MR may provide a new insight into the Trojan horse strategy of McC and opens a new route for antibiotics design. 

## Supplementary Materials

Supplementary materials are available online.
