# Supplementary material for: The Novel Property of Heptapeptide of Microcin C7 in Affecting the Cell Growth of Escherichia coli
Source: Molecules. 2017 Mar 8;22(3):432. doi: 10.3390/molecules22030432 (PMC6155343; doi:10.3390/molecules22030432)
Supplement: Supplementary file 1 [file molecules-22-00432-s001.pdf]

SUPPORTING INFORMATION

Title

The Novel Property of Heptapeptide of Microcin C7 in Affecting the Cell Growth of *E. Coli*

Authors

Rensen Ran, Huan Zeng, Dong Zhao, Ruiyuan Liu and Xia Xu

Contents of supporting information

Methods
Supplemental Figure S1. Basal expression of β-galactosidase *E.coli* BL21.
Supplemental Figure S2. The colony forming units of groups with MR peptide.
References

Methods

1. Basal expression of β-galactosidase assays

Two groups were run to prove the basal expression of β-galactosidase in *E.coli* BL21. One group containing 2mL bacteria solution (1×10^8CFU/mL) and 0.5mL 4mM ONPG solution was incubated for 60 min at 30℃. The bacterial culture was then passed through a 0.22μm filter, dubbed "Bacteria solution". And another group only containing 2mL bacteria solution (1×10^8CFU/mL) was incubated for 60 min at 30℃. Subsequently, the bacterial culture was passed through a 0.22μm filter and the supernatant was added 0.5mL 4mM ONPG solution, and then the solution was incubated for 60 min at 30℃, dubbed "Supernatant". At last, the hydrolysis of ONPG was determined at 420 nm using a spectrophotometer. The experiments were performed in triplicate.

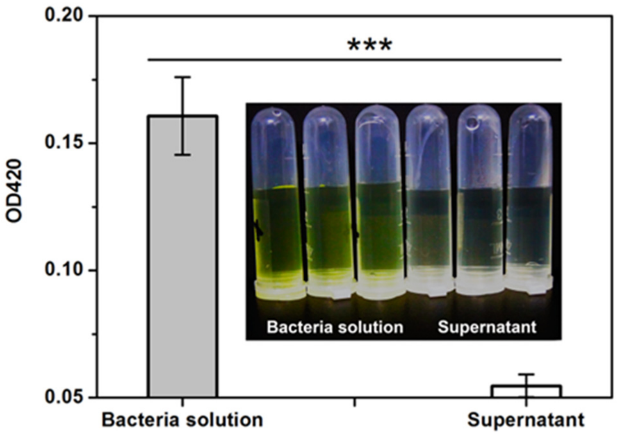

Supplemental Figure S1. The results of basal expression of β-galactosidase assays are represented. ***$\alpha < 0.001$ vs control. The results represent mean values ± SD of three independent experiments.

2. The colony forming units of groups with MR peptide

The role of sublethal concentration of peptides in *E.coli* BL21 growth was tested as the following procedure[1]. The bacteria were prepared using the same procedure

described in the MIC measurement. The samples were treated with peptides at a series of concentration (MIC/2, MIC/4, MIC/8) and incubated at 37℃ for 1.5h under constant shaking (80 rpm). Samples were taken for a series of ten-fold dilutions, and plated out in MHA plates. The plates were incubated over night at 37℃ and the bacteria were counted by CFU.

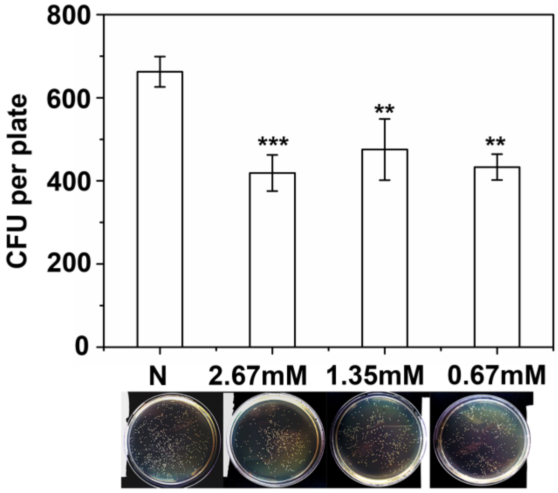

Supplemental Figure S2. The colony forming units of groups with MR peptide is represented. Into each well of a 48-well plate was added 50 μL peptides solution at a series of concentration (MIC/2, MIC/4, MIC/8) and incubated at 37℃ for 1.5 hours. Immediately, samples were taken for a series of ten-fold dilutions, and plated out in MHA plates. The plates were incubated over night at 37℃ and the bacteria was counted by CFU. The result was represented as the percentage of the CFU of groups with peptides in the CFU of control. Control was run without peptides. **$\alpha < 0.01$, ***$\alpha < 0.001$ vs control. The results represent mean values ± SD of three independent experiments.

Reference

1 Xiong, Menghua, et al. "Helical antimicrobial polypeptides with radial amphiphilicity." Proceedings of the National Academy of Sciences 112.43 (2015): 13155-13160.
